# A217 EVALUATING PREDICTIVE SCORING AND OUTCOMES OF PATIENTS DISCHARGED HOME FROM THE EMERGENCY DEPARTMENT WITH UPPER GASTROINTESTINGAL BLEEDING

**DOI:** 10.1093/jcag/gwad061.217

**Published:** 2024-02-14

**Authors:** M Saunders, M Jones, C Lawlor, D Savage, P Zezos

**Affiliations:** Internal Medicine, NOSM University, Sudbury, ON, Canada; Internal Medicine, NOSM University, Sudbury, ON, Canada; Internal Medicine, NOSM University, Sudbury, ON, Canada; Emergency Department, Thunder Bay Regional Health Sciences Centre,, Thunder Bay, ON, Canada; Division of Gastroenterology, Thunder Bay Regional Health Sciences Centre,, Thunder Bay, ON, Canada

## Abstract

**Background:**

Upper gastrointestinal bleed (UGIB) is a common emergency department (ED) presentation. The Glasgow-Blatchford Bleeding Score (GBS) is a validated tool for predicting UGIBs requiring admission for endoscopic evaluation. Utilization of GBS in Northern Ontario EDs may help risk stratify UGIB to decrease return to care.

**Aims:**

This study aimed to evaluate factors and outcomes of patients discharged from the ED with suspected UGIB.

**Methods:**

A total of 1,139 charts were identified with an ICD-10 ED diagnosis of gastrointestinal hemorrhage from 2016-2022. Of these, 92 charts met inclusion criteria. Patients admitted to hospital from the index ED visit, who were below the age of 18 years at the time of presentation, or with clinically suspected lower gastrointestinal hemorrhage were excluded. Patient were further grouped by place of residence if their primary address was within Thunder Bay city limits or not.

**Results:**

There were 48 males (52%) with a median age of 58.7 (IQR 32.2) at presentation with UGIB to ED. The median (IQR) Charlson Comorbidity Index (CCI) and GBS were 3 (5) and 3 (6), respectively. The odds ratio for returning to the ED and being admitted for UGIB within sixty days was 4.07 times greater for patients from the region compared to Thunder Bay residents (95% CI 1.28–12.97). GBS was found to be significantly greater (*p*=0.048, Mann-Whitney U-Test) among seven patients who underwent emergent endoscopy from the ED and were discharged home. In univariate K-M survival analysis, the mean survival time to hospital admission at sixty days was 45 days among those with a presenting GBS score of ampersand:003E3, compared to 57 days when GBS was 3 or less (*p*ampersand:003C0.005, log rank Mantel-Cox). After adjustment for age, sex, place of residence, and CCI, there was a 17% increase in the expected hazard for hospital admission at sixty days for each point increase in GBS (95% CI 1.03–1.34). Similarly, the expected hazard for follow-up hospital admission was 2.95 times higher (95% CI 1.11–7.85) in patients living outside Thunder Bay travelling to seek care. There were no statistically significant hazards identified between age, sex, or CCI and hospital admission (Cox proportional hazards model, p=0.001). Of patients admitted to hospital within sixty days of ED discharge (n=17), the median length of stay was five days with 56% requiring blood transfusion and 83% receiving endoscopy.

**Conclusions:**

Higher GBS scores and residence outside of Thunder Bay increase the likelihood of revisit and admission to hospital for UGIB following ED discharge. Future research will aim to compare outcomes among larger urban centers.

**Funding Agencies:**

NOAMA

